# Bispecific antibodies combined with chemotherapy in solid tumor treatment, the path forward?

**DOI:** 10.3389/fimmu.2025.1568724

**Published:** 2025-04-25

**Authors:** Yici Yan, Jing Yuan, Yanyang Peng, Chenxi Zhou, Xinbo Liu, Leitao Sun, Qiaoling Song

**Affiliations:** ^1^ The First Affiliated Hospital of Zhejiang Chinese Medical University (Zhejiang Provincial Hospital of Chinese Medicine), Hangzhou, China; ^2^ School of Humanities and Management, Zhejiang Chinese Medical University, Hangzhou, China; ^3^ Academy of Chinese Medical Science, Zhejiang Chinese Medical University, Hangzhou, China; ^4^ Key Laboratory of Neuropharmacology and Translational Medicine of Zhejiang Province, School of Pharmaceutical Sciences, Zhejiang Chinese Medical University, Hangzhou, China

**Keywords:** bispecific antibody, chemotherapy, solid tumor, efficacy, safety, meta-analysis

## Abstract

**Background:**

Bispecific antibodies (bsAbs) introduced a novel strategy in anticancer therapy when chemotherapy alone could not meet life expectancy. Nonetheless, the efficacy of monotherapy was limited, and the safety profile of bsAbs combined with chemotherapy remained uncertain.

**Methods:**

Literature retrieval was carried out through PubMed, Embase, and Cochrane from inception to January, 2025. Progression-free survival (PFS), overall survival (OS), and overall response rate (ORR), along with adverse effects (AEs), were utilized to assess the efficacy and safety. Publication bias was calculated using Funnel plots and Egger’s test. Heterogeneity was examined through subgroup and sensitivity analyses. The protocol was preregistered in the International Prospective Register of Systematic Reviews (CRD42025633628).

**Results:**

A total of 8 eligible clinical studies with 2,495 patients were included. Compared with chemotherapy alone, bsAb+chemotherapy exhibited positive outcomes in PFS (hazard ratio (HR): 0.52; 95% confidence interval (CI): 0.44-0.60; p<0.01), OS (HR: 0.67, 95% CI: 0.57-0.77; p<0.01), and ORR (HR: 0.31, 95% CI: 0.16-0.47; p<0.01). Subgroup analysis revealed that female patients, Asian patients, those under 65 years of age, and patients treated with IgG-like bsAb were more likely to benefit from the survival advantages of bsAb+chemotherapy. Despite the occurrence of leukopenia, metabolism-related, and skin-related AEs, RR of AEs in other systems showed no statistical significance.

**Conclusion:**

BsAb+chemotherapy was superior to chemotherapy alone, especially in female patients, Asian patients, those under 65 years of age, and patients receiving IgG-like bsAb. Additionally, while the AEs associated with bsAb+chemotherapy are generally manageable, there is still room for improvement.

**Systematic review registration:**

https://www.crd.york.ac.uk/prospero/, identifier CRD42025633628.

## Introduction

According to the latest estimates by GLOBOCAN, in 2022 the annual number of solid tumors globally reached 18.7 million, accounting for over 90% of all cancer cases globally (1). In the same year, approximately 9.7 million deaths were caused by solid tumors and the number continues to rise steadily. Chemotherapy has long been the backbone of treatment for solid tumors. However, chemotherapy alone is often limited by off-target toxicity, drug resistance, and immunosuppression, underscoring the need for more targeted and effective therapeutic strategies.

Bispecific antibodies (bsAbs) emerge as a game-changing approach in anticancer therapy by simultaneously binding to two antigens or two epitopes of the same antigen (2). This dual targeting capability enables bsAbs to bridge immune cells, such as T cells or natural killer (NK) cells, with tumor cells, facilitating immune cell activation and tumor elimination. BsAbs can be categorized into IgG-like and non-IgG-like formats. IgG-like bsAbs retain Fc regions, enabling effector functions like antibody-dependent cellular cytotoxicity (ADCC) and complement-dependent cytotoxicity (CDC), while non-IgG-like bsAbs often lack Fc regions, favoring smaller size and improved tissue penetration. By engaging multiple tumor-associated targets, bsAbs can enhance precision in tumor targeting, overcome tumor heterogeneity, and counteract immune evasion mechanisms (3). Additionally, bsAbs can be engineered to address key challenges in cancer treatment, such as drug resistance and the immunosuppressive tumor microenvironment. Beyond their standalone efficacy, bsAbs are increasingly explored in combination with chemotherapy or other immunotherapies, offering the potential for longer-lasting disease control, improved survival outcomes, and the ability to overcome resistance observed with monotherapy. Although these new therapies provide additional options, they also carry specific and potential toxicities for patients. Most notable is the withdrawal from the European market of catumaxomab in 2017 (4).

To date, 11 bsAbs have been approved by the Food and Drug Administration (FDA), European Medicines Agency (EMA) or National Medical Products Administration (NMPA) for cancer treatment (5). However, the majority of these approvals are for hematologic malignancies, with only a handful target solid tumors (5). This may be explained by the poor penetration and trafficking of bsAbs, the inherent complexity of the solid tumor microenvironment, and the prevalence of immune evasion mechanisms in solid tumors (6, 7). Despite these challenges, bsAbs for solid tumors is predicted to have substantial market potential due to its wide mass foundation.

Overall, bsAbs+chemotherapy seems to be the path forward in the treatment of solid tumors. However, to the best of our knowledge, no systematic analysis has yet been conducted to substantiate this conclusion, particularly in comparison with the hematologic malignancies (8, 9). Furthermore, existing randomized control trails (RCTs) involve different kinds of bsAbs, various sample sizes, and diverse tumor types. Therefore, a meta-analysis of published RCTs was performed. The main objective of this study is to evaluate the efficacy and safety of bsAbs+chemotherapy for patients with solid tumors.

## Methods

### Literature search strategy

This meta-analysis was conducted following the Preferred Reporting Items for Systematic Reviews and Meta-Analyses (PRISMA) guidelines. A thorough search was conducted on three databases, including PubMed, Embase, and Cochrane Library, from inception to January 2, 2025 by two independent investigators. Additional records identified through other sources including ClinicalTrials.gov, American Society of Clinical Oncology (ASCO), European Society for Medical Oncology (ESMO) and American Association for Cancer Research (AACR). Reference lists were reviewed for completeness to avoid missing relevant articles. Both MeSH terms and free terms were used. The MeSH terms used were as follows: “Bispecific Antibodies” and “Neoplasms”. The detailed search strategy in PubMed is presented in **Supplementary Table 1**. The protocol was preregistered in the International Prospective Register of Systematic Reviews (CRD42025633628).

### Inclusion and exclusion criteria

The PICOS criteria were as follows: (1) Participants were patients with diagnosed solid tumor; (2) Intervention group was patients treated with bispecific antibody plus chemotherapy treatment; (3) Control group was patients treated with chemotherapy with or without placebo. (4) Outcomes included overall survival (OS) or progress-free survival (PFS), with or without overall response rate (ORR) and adverse events (AEs); (5) Study type was randomized clinical trials (RCTs).

The exclusion criteria were as follows: (1) studies that not reported specific data, including hazard ratio (HR) along with corresponding 95% confidence interval (CIs); (2) studies in which patients were diagnosed with hematological tumors; (3) studies without full-text; (4) studies that were single arms, reviews, observational studies, case reports, meta-analyses, letters, comments.

Two investigators independently lay down the inclusion and exclusion criteria. Any discrepancy would be addressed among three investigators.

### Data extraction

Data were independently extracted and cross-checked by two investigators. The following characteristic information of the included studies was recorded: (1) Study characteristic: first author, publication year, location, follow-up, cancer type, intervention group, control group, phase, line, sample size, median PFS and median OS, and drug target; (2) Study outcomes: effect estimates of OS, PFS, ORR, and AEs of all grade and ≥grade 3.

### Quality assessment

Two researchers used the Cochrane Collaboration’s tool to independently assess the quality of included RCTs. Briefly, each article was evaluated across 7 domains, including bias arising from intended the randomization process, bias due to allocation concealment, bias due to blinding of participants and personnel, bias due to blinding of outcome assessment, bias due to incomplete outcome data, bias due to selective reporting, and other bias. Each domain was judged as “low risk,” “high risk,” or “unclear risk” based on the criteria outlined in the Cochrane Handbook. Any discrepancies in their judgments were resolved through discussion and consensus.

### Statistical analysis

Statistical analyses of study outcomes were performed and pooled as forest plots by Stata 18.0. The HR with 95% CI was used to assess the outcomes PFS and OS. HR<1 favored the intervention group, while HR>1 favored the control group. The Relative Risk (RR) with 95% CI was used to analyze ORR and AEs. For ORR and AEs, RR<1 indicated that the control group had a higher response rate and toxicity, while RR>1 indicated the opposite. Chi-square Q test and I^2^ statistic was used to detect statistical heterogeneity. I^2^<30% indicated low heterogeneity, 30%≤I^2^ ≤ 60% represented moderate heterogeneity, and I^2^>60% revealed high heterogeneity. Due to the clinical heterogeneity from diversity of tumor types and difference in intervention, the random‐effects model was used for combined analysis. Furthermore, subgroup analysis was implemented to identify the factors contributing risk of bias. We also conducted the sensitivity analysis by sequential exclusion of included individual trial. Funnel plots and Egger’s tests were also used to examine potential publication bias. All reported P-values were two-sided, with statistical significance defined as p<0.05.

## Result

### Literature search results

**Figure 1:**
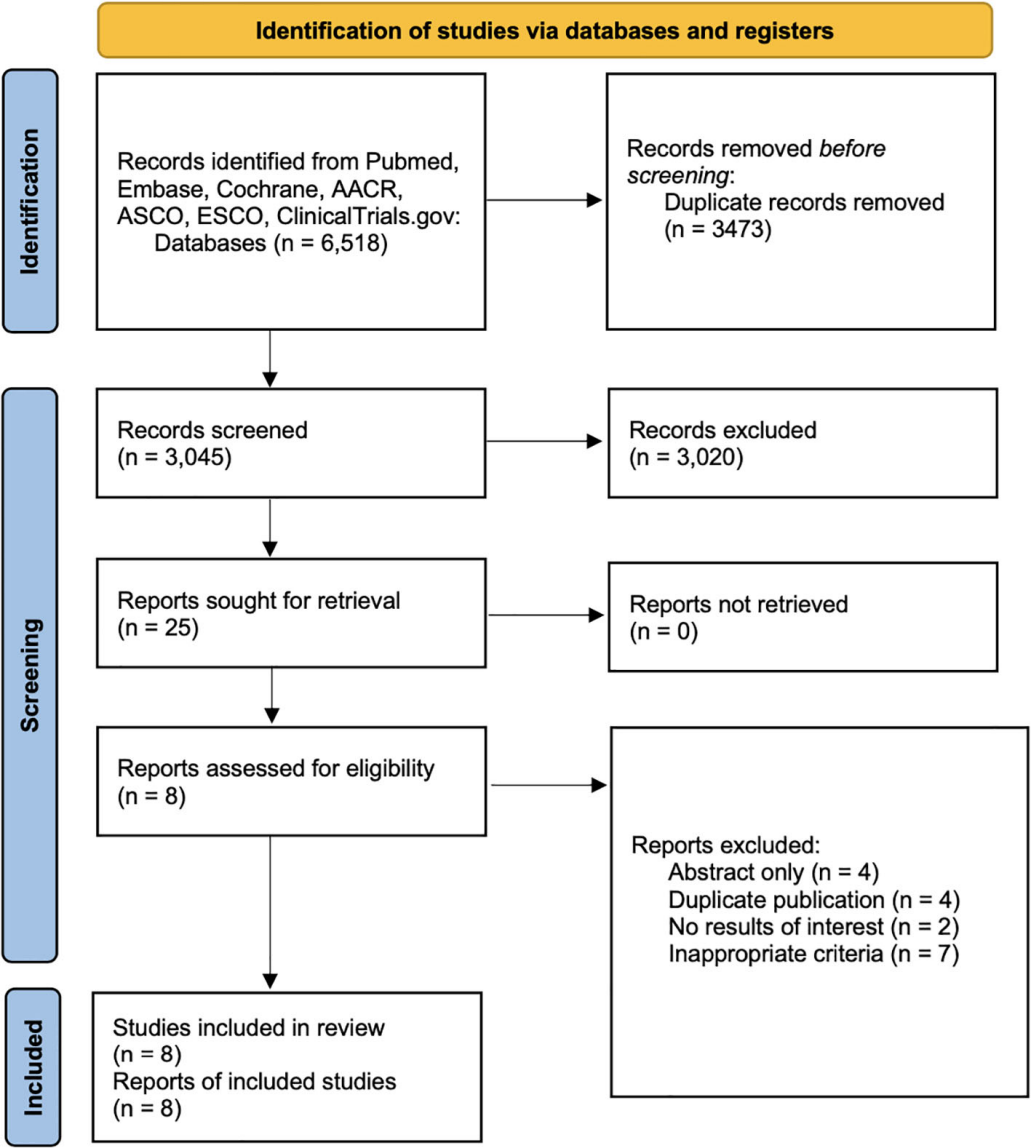
Flowchart of the study selection.

A total of 6,518 relevant articles were initially retrieved, and after removing duplicates, 3,045 articles remained. A preliminary review of titles, abstracts, and keywords led to the exclusion of 3,020 articles. The comprehensive reviews of the 25 surviving articles that might have qualified for inclusion were then conducted. Adhering to a rigorous screening process predicated on predetermined inclusion and exclusion criteria, 17 articles were excluded due to no results of interest, inappropriate criteria, duplicates, or no full-text available. Finally, 8 articles were deemed eligible and included in the meta-analysis (10–17). The detailed selection process is illustrated in **Figure 1**.

### Basic characteristics of included studies

A total of 2,495 patients were enrolled in our study. The publication year ranged from 2018 to 2024, originating from Germany, Canada, The United States, and China. Among 8 eligible articles, four were conducted in single center and the remaining four were in multi-center. Five were used as 1 line therapy, one was performed as 1/2 line therapy, one was used as ≥2 line therapy. The median follow-up period ranged from 7.9 to 52.0 months. Overall, seven cancer types were identified in this review, incorporating gastric cancer (GC), peritoneal cancer (PC), metastatic pancreatic cancer, non-small cell lung cancer (NSCLC), gastric or gastroesophageal junction (G/GEJ) adenocarcinoma, biliary tract cancer, and cervical cancer (CC). The combination regimen included catumaxomab+5-fluorouracil, leucovorin, oxaliplatin, docetaxel (FLOT), istiratumab+nab-paclitaxel plus gemcitabine regime (NG), amivantamab+carboplatin plus pemetrexed regimen (CP), cadonilimab+capecitabine plus oxaliplatin regimen (XELOX), ivonescimab+CP, bintrafusp alfa+gemcitabine plus cisplatin regimen (GemCis), cadonilimab+cisplatin plus paclitaxel regimen (GP)/paclitaxel plus carboplatin regimen (PCb). The detailed characteristics of included studies were shown in **Table 1**; **Supplementary Table 2**.

**Table 1:** Characteristic of included studies.

Author, year	Country	Design	Line^1^	Cancer type	Follow-up, months	Sample size (M/F)	Race (non-A/A)	I/C	No. of patients	Median PFS, months	Median OS, months
Knödler M, 2018 (10)	Germany	Single center	NA	GC and PC	52	31 (17/14)	31/0	I: Catumaxomab+ FLOT	15	6.7	13.2
								C: FLOT	16	5.4	13.0
Kundranda M, 2020 (11)	Canada	Multi-center	1	Metastatic pancreatic cancer	NA	88 (46/42)	88/0	I: Istiratumab+NG	43	3.6	8.9
								C: Placebo+NG	45	7.3	11.7
Zhou C, 2023 (12)	US	Multi-center	1	NSCLC	14.9	308 (130/178)	117/186	I: amivantamab+CP	153	11.4	NA
								C: CP	155	6.7	24.4
Ji J, 2024 (17)	China	Single center	1	G/GEJ adenocarcinoma	18.6	610 (474/136)	0/610	I: Cadonilimab+XELOX	305	7.0	15.0
								C: Placebo+XELOX	305	5.3	10.8
Fang W, 2024 (13)	China	Single center	≥2	NSCLC	7.9	322 (156/166)	0/322	I: Ivonescimab+CP	161	7.2	NA
								C: Placebo+CP	161	7.1	NA
Oh DY, 2024 (14)	US	Multi-center	1	Biliary tract cancer	18.7	297 (151/146)	114/183	I: Bintrafusp alfa+GemCis	73	5.5	11.5
								C: Placebo+GemCis	77	5.6	11.5
Passaro A, 2024 (15)	US	Multi-center	1/2	NSCLC	8.7	394 (238/156)	204/190	I: Amivantamab+CP	131	8.2	6.3
								C: CP	263	4.2	4.2
Wu X, 2024 (16)	China	Single center	1	CC	25.6	445 (0/445)	0/445	I: Cadonilimab+GP/PCb (+bevacizumab)	222	12.7	NA
								C: Placebo+GP/PCb (+bevacizumab)	223	8.1	22.8

US, the United States; NA, no available; GC, gastric cancer; PC, peritoneal carcinoma; NSCLC, non-small cell lung cancer; G/GEJ, gastric or gastroesophageal junction; CC, cervical cancer; I, intervention group; C, control group; FLOT, 5-fluorouracil, leucovorin, oxaliplatin, docetaxel; NG, nab-paclitaxel plus gemcitabine regimen; CP, carboplatin plus pemetrexed regimen; XELOX, capecitabine plus oxaliplatin regimen; GemCis, gemcitabine plus cisplatin regimen; GP, cisplatin plus paclitaxel regimen; PCb, paclitaxel plus carboplatin regimen; PFS, progress-free survival; OS, overall survival; M, male; F, female; non-A, non-Asian people; A, Asian people.

^1^treatment line.

### Efficacy

**Figure 2:**
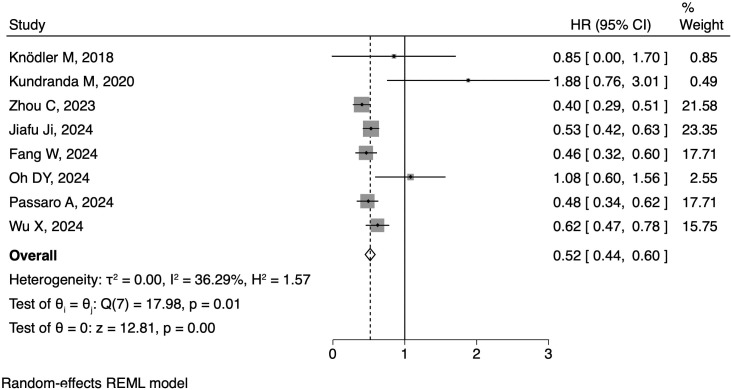
Forest plot of the meta-analysis on PFS.

**Figure 3:**
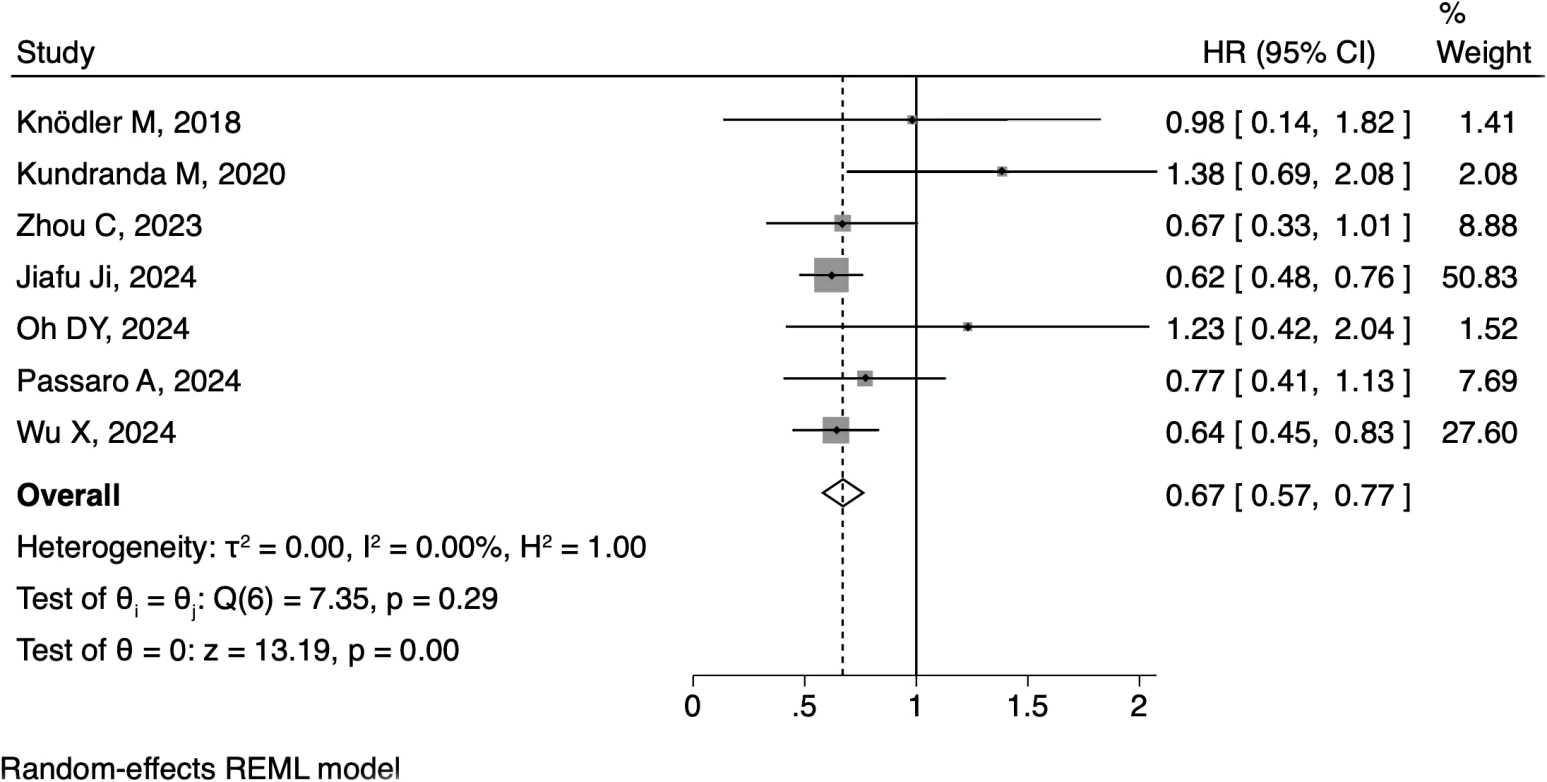
Forest plot of the meta-analysis on OS.

**Figure 4:**
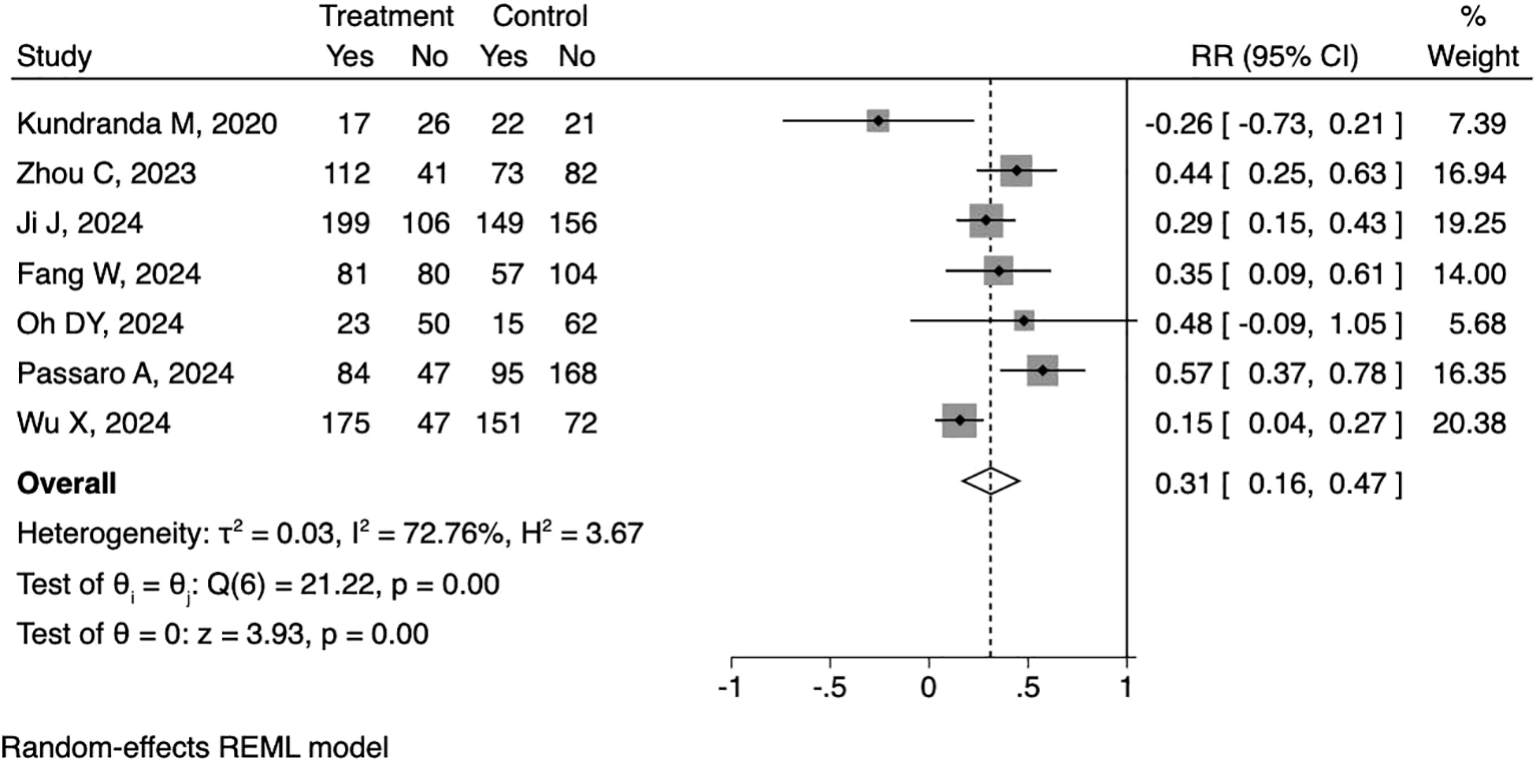
Forest plot of the meta-analysis on ORR.

All of the eight articles reported HRs as PFS outcome. The pooled HR for PFS was 0.52 (95% CI: 0.44-0.60, **Figure 2**), with statistical significance (p<0.01) and moderate heterogeneity (I^2^ = 36.29%). As for OS outcome, seven articles reported corresponding HRs. The pooled HR for OS was 0.67 (95% CI: 0.57-0.77, **Figure 3**), along with statistical significance (p<0.01) and low heterogeneity (I^2^ = 0.0%). Seven articles reported ORR data, with a positive outcome (RR: 0.31; 95% CI: 0.16-0.47; p<0.01, **Figure 4**).

### Subgroup analysis

Subgroup analysis was conducted to make a further exploration of combination regimen, mainly on age, brain metastasis, bsAb format, cancer type, Eastern Cooperative Oncology Group (ECOG) Performance Status (PS), metastasis, race, sex, and weight (**Table 2**).

**Table 2:** Subgroup analysis of PFS and OS.

Subgroup			PFS					OS		
	No. of studies	No. of cases		HR (95% CI)	p^1^	No. of studies	No. of cases		HR (95% CI)	p^1^
Age										
<65 yrs	5	1,174		0.54 (0.39, 0.74)	<0.01	5	482		0.64 (0.49, 0.83)	<0.01
≥65 yrs	5	592		0.61 (0.40, 0.92)	0.02	5	425		0.97 (0.34, 2.76)	0.96
Brain metastasis										
Yes	3	321		0.52 (0.39, 0.69)	<0.01	NA	NA		NA	NA
No	3	703		0.42 (0.33, 0.54)	<0.01	NA	NA		NA	NA
bsAb format										
Non-IgG-like	2	328		1.03 (0.70, 1.52)	0.88	2	328		1.13 (0.69, 1.83)	0.63
IgG-like	6	2,167		0.57 (0.44, 0.75)	<0.01	5	1,845		0.74 (0.58, 0.93)	0.01
Cancer type										
GC	2	641		0.55 (0.43, 0.69)	<0.01	2	641		0.67 (0.48, 0.92)	0.01
NSCLC	3	1,024		0.44 (0.38, 0.53)	<0.01	2	1,024		0.72 (0.52, 1.00)	0.05
ECOG PS										
0	5	617		0.60 (0.36, 1.00)	0.05	2	610		1.03 (0.40, 2.67)	0.95
1	5	1,149		0.54 (0.45, 0.66)	<0.01	2	297		0.72 (0.41, 1.26)	0.25
Race										
Asian	6	1,936		0.57 (0.44, 0.73)	<0.01	3	1,238		0.64 (0.54, 0.76)	<0.01
Non-Asian	4	466		0.59 (0.38, 0.91)	0.02	2	145		1.77 (0.42, 7.39)	0.44
Sex										
Female	5	1,091		0.53 (0.39, 0.73)	<0.01	3	1,065		0.70 (0.55, 0.89)	<0.01
Male	4	675		0.63 (0.38, 1.03)	0.06	2	287		0.97 (0.31, 3.00)	0.95
Weight										
<80 kg	2	599		0.46 (0.37, 0.57)	<0.01	NA	NA		NA	NA
≥80 kg	2	103		0.38 (0.19, 0.70)	<0.01	NA	NA		NA	NA

yrs, years; GC, gastric cancer; NSCLC, non-small cell lung cancer; ECOG PS, Eastern Cooperative Oncology Group Performance Status; No, number; PFS, progression-free survival; HR, hazard ratio; OS, overall survival; NA, not available.

^1^p<0.05 indicates significant.

BsAb+chemotherapy benefits patients both under (HR: 0.54; 95% CI: 0.39-0.74; p<0.01) or above the age of 65 years (HR: 0.61; 95% CI: 0.40-0.92; p=0.02) in terms of PFS. In terms of OS, bsAb+chemotherapy benefits patients under the age of 65 (HR: 0.64; 95% CI: 0.49-0.83; p<0.01), but for those above the age of 65, no marked survival benefit was observed (p=0.96). Totally, three articles focused on brain metastasis, and both metastasis group (HR: 0.52; 95% CI: 0.39-0.69; p<0.01) and non-metastasis group (HR: 0.42; 95% CI: 0.33-0.54; p<0.01) confirmed the superior PFS-related efficacy of bsAb+chemotherapy. As for bsAb format, both PFS-related (95% CI: 0.44-0.75; p<0.01) and OS-related benefit (95% CI: 0.58-0.93; p=0.01) were observed in patient treated with IgG-like bsAb (istiratumab, amivantamab, cadonilimab, ivonescimab)+chemotherapy. However, bsAb+chemotherapy failed to achieve better OS (p=0.63) or PFS (p=0.88) in patients receiving non-IgG-like bsAb (catumaxomab, bintrafusp alfa). When stratified by cancer type, two articles investigated on GC (HR: 0.55; 95% CI: 0.43-0.69; p<0.01) and three were on NSCLC (HR: 0.44; 95% CI: 0.38-0.53; p<0.01). Both cancer types exhibited statistical significance on PFS outcome. In terms of ECOG PS, PFS-related benefits were observed in patients with ECOG PS=1 (HR: 0.54; 95% CI: 0.45-0.66; p<0.01). No statistical difference was observed in OS benefit regarding ECOG PS (ECOG PS=1 (p=0.25), ECOG PS=0 (p=0.95)). When stratified by race, both Asian group (HR: 0.57; 95% CI: 0.44-0.73; p<0.01) and non-Asian group (HR: 0.59; 95% CI: 0.38-0.91; p=0.02) demonstrated PFS benefits. In terms of OS, Asian group (HR: 0.64; 95% CI: 0.54, 0.76; p<0.01) showed survival benefits, but there is no statistical difference between bsAb+chemotherapy and chemotherapy for non-Asian group (p=0.44). Regarding sex, a synthesized estimate from five studies on female indicated better prognosis on patients with bsAb+chemotherapy in terms of PFS (HR: 0.53; 95% CI: 0.39-0.73; p<0.01) and OS (HR: 0.70; 95% CI: 0.55-0.89; p<0.01), while male group failed to exhibit therapeutic superiority in terms of PFS (p=0.06) and OS (p=0.95). When stratified by weight, PFS-related benefit was observed in both <80 kg group (HR: 0.46; 95% CI: 0.37-0.57; p<0.01) and ≥80 kg group (HR: 0.38; 95% CI: 0.19-0.70; P<0.01).

### Safety

The safety profile of combination regimen was illustrated in **Table 3**; **Supplementary Table 3**. It was carried out in digestive system, hematological system, liver function, metabolism, renal function, skin, and others. When exploring the incidence of severe side effects, we subsequently performed high-grade AEs (grade≥3).

**Table 3:** Treatment-related common adverse events in this meta-analysis.

Adverse events		RR (95% CI)					
		No. of studies	All Grade	P value	No. of studies	Grade≥3	P value
Digestive system	Abdominal pain	2	0.73 (0.35, 1.52)	0.40	2	0.32 (0.06, 1.71)	0.18
	Constipation	6	1.13 (0.76, 1.69)	0.55	2	1.38 (0.09, 21.35)	0.82
	Diarrhea	5	1.60 (0.93, 2.74)	0.09	5	1.72 (0.71, 4.15)	0.23
	Nausea	6	1.02 (0.73, 1.42)	0.91	5	1.14 (0.42, 3.14)	0.80
	Stomatitis	4	3.26 (0.97, 10.92)	0.06	4	2.36 (0.49, 11.33)	0.28
	Vomiting	7	1.22 (0.88, 1.68)	0.24	7	2.15 (0.87, 5.30)	0.10
Hematological system	Anemia	7	1.10 (0.86, 1.41)	0.43	7	0.93 (0.76, 1.15)	0.52
	Leukopenia	3	1.40 (0.66, 2.98)	0.38	3	2.31 (1.47, 3.63)	<0.01
	NE decrease	2	0.67 (0.35, 1.31)	0.24	2	0.61 (0.27, 1.34)	0.21
	Neutropenia	6	1.07 (0.82, 1.40)	0.60	6	1.29 (0.91, 1.83)	0.15
	PLT decrease	6	1.06 (0.72, 1.56)	0.77	5	0.98 (0.50, 1.92)	0.95
	WBC decrease	3	0.89 (0.73, 1.09)	0.26	3	0.83 (0.51, 1.36)	0.46
Liver function	ALT increased	4	1.54 (0.81, 2.94)	0.27	4	1.44 (0.75, 2.76)	0.27
	AST increased	4	1.54 (0.79, 3.02)	0.20	4	1.50 (0.53, 4.26)	0.44
	GGT	2	2.67 (0.82, 8.72)	0.10	2	1.52 (0.22, 10.26)	0.67
Metabolism	HYPE	3	3.23 (1.19, 8.77)	0.02	2	7.81 (1.33, 45.91)	0.02
	HyperG	2	2.33 (0.97, 5.59)	0.06	2	3.29 (1.05, 10.35)	0.04
	HypoK	5	1.65 (1.09, 2.48)	0.02	5	2.07 (1.07, 4.00)	0.03
Renal function	P-Edema	3	3.86 (0.60, 24.73)	0.15	3	3.35 (0.62, 18.10)	0.16
	Proteinuria	2	1.34 (0.98, 1.83)	0.06	2	1.30 (0.43, 3.91)	0.64
Skin	DA	2	7.46 (4.20, 13.26)	<0.01	2	16.51 (2.16, 126.29)	0.01
	Paronychia	2	23.02 (2.42, 218.70)	0.01	2	16.95 (2.20, 130.80)	0.01
	Pyrexia	5	1.78 (0.79, 4.01)	0.17	3	1.00 (0.17, 5.90)	1.00
	Rash	6	3.25 (1.90, 5.54)	<0.01	5	9.35 (3.05, 28.64)	<0.01
Others	Asthenia	4	1.84 (1.10, 3.06)	0.02	4	1.38 (0.57, 3.30)	0.48
	Fatigue	7	1.09 (0.71, 1.68)	0.70	7	0.84 (0.41, 1.73)	0.64
	IRR	4	25.15 (6.82, 92.78)	<0.01	3	15.53 (2.91, 82.83)	<0.01
	Weight decreased	3	1.42 (1.02, 1.98)	0.04	3	2.89 (0.78, 10.67)	0.11

NE, neutrophil; PLT, platelet; WBC, white blood cell; ALT, alaninetransaminase; AST, aspartate aminotransferase; GGT, γ-glutamyltransferase; HYPE, hypoproteinemia; HyperG, hyperglycemia; HypoK, hypokalemia; P-Edema, peripheral edema; DA, dermatitis acneiform; IRR, infusion-related reaction; RR, relative risk.

RR of all grade AEs of digestive system revealed no statistical significance: abdominal pain (p=0.40), constipation (p=0.55), diarrhea (p=0.09), nausea (p=0.91), stomatitis (p=0.06), and vomiting (p=0.24). RR of grade ≥3 AEs of digestive system also showed no statistical significance: abdominal pain (p=0.18), constipation (p=0.82), diarrhea (p=0.23), nausea (p=0.80), stomatitis (p=0.28), and vomiting (p=0.10). In hematological system, despite leukopenia (RR: 2.31, 95% CI: 1.47-3.63; p<0.01), RR of grade ≥3 AEs revealed no statistical significance: anemia (p=0.52), neutrophil decrease (p=0.21), neutropenia (p=0.15), platelet decrease (p=0.95), and white blood cell (WBC) decrease (p=0.46). RR of AEs of all grade revealed no statistical significance: anemia (p=0.43), leukopenia (p=0.38), neutrophil decrease (p=0.24), neutropenia (p=0.60), platelet decrease (p=0.77), and WBC decrease (p=0.26). No statistical significance in AEs of all grade or grade ≥3 was found in liver function and renal function. In metabolism, higher incidence of hypoproteinemia (RR: 3.23; 95% CI: 1.19-8.77; p=0.02) and hypokalemia (RR: 1.65; 95% CI: 1.09-2.48; p=0.02) was observed in all grade AEs. When it comes to severe metabolic disorders (grade ≥3), elevated incidence of hypoproteinemia (RR: 7.81; 95% CI: 1.33-45.91; p=0.02), hyperglycemia (RR: 3.29; 95% CI: 1.05-10.35; p=0.04) and hypokalemia (RR: 2.07; 95% CI: 1.07-4.00; p=0.03) was identified in combination arm. As for skin-related AEs, higher incidence of dermatitis acneiform (RR: 7.46; 95% CI: 4.20, 13.26; p<0.01), paronychia (RR: 23.02; 95% CI: 2.42-218.70; p=0.01), and rash (RR: 3.25; 95% CI: 1.90-5.54; p<0.01) was found in all grade AEs. When it comes to severe (grade ≥3) skin toxicity, patients with combination treatment tended to have elevated risk of dermatitis acneiform (RR: 16.51; 95% CI: 2.16-26.29; p=0.01), paronychia (RR: 16.95; 95% CI: 2.20-130.80; p=0.01) and rash (RR: 9.35; 95% CI: 3.05-28.64; p<0.01). Additionally, increments in asthenia (RR: 1.84; 95% CI: 1.10, 3.06; p=0.02), infusion-related reaction (RR: 25.15; 95% CI: 6.82-92.78; p<0.01), and weight decreased (RR: 1.42; 95% CI: 1.02-1.98; p=0.04) were observed in all grade AEs. And for grade ≥3 AEs, incidence of severe infusion-related reaction (RR: 15.53; 95% CI: 2.91-82.83; p<0.01) tended to be elevated. No statistical significance in grade ≥3 AEs was found in asthenia (p=0.48), fatigue (p=0.64) and weight decreased (p=0.11).

### Quality assessment

The individual evaluation of each article included in this meta-analysis is depicted in **Supplementary Figure 1**; **Figure 2**. Seven articles showed a low risk of bias while one was considered as moderate reliability, specifically in domain D5.

### Publication bias and sensitivity analysis

The funnel plots on PFS (**Supplementary Figure 3**) and ORR (**Supplementary Figure 4**) were symmetrical, suggesting no signs of publication bias. And the one on OS outcome was slightly asymmetrical (**Supplementary Figure 5**), indicating a potential presence of publication bias. Egger’s test was performed to further assess publication bias. No significant publication bias was observed for PFS (p = 0.111) or ORR (p = 0.567). A statistically significant Egger’s test result (p = 0.047) suggested the presence of potential publication bias for OS. Sensitivity analysis was performed to evaluate the reliability of the findings. No statistically significant changes in the overall results were observed after removing each included study, thus confirming the reliability and validity of our findings.

## Discussion

This is the first meta-analysis to show that adding bsAbs to chemotherapy significantly and clinically meaningfully improved PFS and OS in solid tumors. In addition, treatment with bsAbs+chemotherapy was associated with a higher ORR, which led to a longer duration of response than with chemotherapy alone. Mechanistically, bsAbs can simultaneously engage multiple tumor-associated targets, overcoming resistance mechanisms that rely on specific molecular alterations within the tumor (5). Chemotherapy, in turn, provides activity against other resistance mechanisms that are independent of these specific pathways (18). When combined, this approach offers broad coverage against the diverse and polyclonal resistance that emerges as the tumor progresses, thereby enhancing the overall therapeutic effectiveness.

As a new kind of immunotherapy, bsAbs has achieved significant success in the field of hematologic malignancies like leukemia and lymphoma, attaining survival rates that were once considered unreachable (19–21). Nevertheless, according to the International Agency for Research on Cancer, solid tumor occurrences constituted over 90% of all cancer diagnoses, significantly surpassing the rates of leukemia and lymphoma (1). Unfortunately, the bsAbs which are effective for leukemia and lymphoma have exhibited unexpectedly low clinical response rates and unsatisfactory efficacy in treating solid tumors featuring specific microenvironments in tumor tissues (22). Although the clinical outcome of bsAbs is less favorable in solid tumors when compare with hematologic malignancies (23, 24), an increasing number of bsAbs targeted solid tumors have been approved and abundant clinical trials are underway. Presently, the main challenges for bsAbs in solid tumors are tumor microenvironment complexity and immune evasion (25). Concretely speaking, while hematologic tumors involve targets expressed on B-cells or bone marrow cells, T-cell-mediated damage to these cells is reversible because hematopoietic stem cells can replenish the lost cells, minimizing systemic impact. Solid tumors, however, are expressed on normal cells, and if T cells kill them, they will cause irreversible damage to the body’s function. Additionally, cold tumors present a further obstacle, as their dense extracellular matrix forms a physical barrier that prevents immune cell infiltration (26). Moreover, immunosuppressive cytokines such as TGF-β and CXCL12 in the tumor microenvironment inhibit T-cell penetration and activity (27), further hindering the effectiveness of bsAbs in these tumors. Chemotherapy can play a crucial role in overcoming these challenges and enhancing the effectiveness of bsAbs in solid tumors. Chemotherapy has been shown to modify the tumor microenvironment in ways that can make it more responsive to immune-based therapies like bsAbs (28). Specifically, chemotherapy can reduce the tumor cell burden, improve vascularization, and help normalize the tumor vasculature, facilitating better immune cell infiltration. This normalization of the microenvironment can reduce the physical barriers, such as the dense extracellular matrix, that typically prevent immune cells from effectively reaching and attacking the tumor (29). Additionally, chemotherapy can induce immunogenic cell death (ICD), which releases tumor antigens and enhances the presentation of these antigens by dendritic cells (30). This process primes the immune system, making the tumor more recognizable to T cells and increasing the potential for immune-mediated tumor destruction. Together, chemotherapy and bsAbs may work synergistically to overcome the key obstacles posed by the tumor microenvironment, offering a promising strategy to improve clinical outcomes in solid tumors. While challenges remain, ongoing research and clinical trials continue to explore ways to refine and optimize this combination approach, with the potential to significantly improve survival rates for patients with solid tumors (31).

Through subgroup analysis, female patients with solid tumors demonstrated better survival outcomes when receiving bsAbs+chemotherapy, which corroborates the findings of Thieblemont C (32) and Michael J (20). They found subgroup involving female showed a trend toward a higher percentage with a complete response. This may be explained by the generally stronger immune responses in females, attributed to hormonal influences (33). Mechanistically, estrogen enhances immune cell activity through multiple pathways: it promotes the proliferation and activation of T cells by upregulating the expression of cytokines such as IL-2 and IFN-γ; enhances the antigen-presenting capacity of dendritic cells by increasing the expression of co-stimulatory molecules like CD80 and CD86; and boosts the cytotoxic activity of natural killer (NK) cells by upregulating perforin and granzyme production (34, 35). Additionally, due to a higher body fat percentage, certain chemotherapy agents are metabolized differently in women, which may optimize the synergistic effect when combined with bsAbs, leading to better therapeutic outcomes (2, 36). As for races, our study suggested Asian patients with solid tumors experienced better survival benefits when treated with bsAbs+chemotherapy. Ethnic differences in somatic mutations such as STK11, TP53 and EGFR may account for the differences of outcome for Asian and non-Asian patients receiving immunotherapy (37). For example, the mutation rate of STK11 differs among Asian (1.6%) and non-Asian patients (12.3%), which was reported previously to affect efficacy of immune checkpoint inhibitors (38, 39). Additionally, ethnicity may act as a key factor that influence the metabolism of chemotherapy agents and monoclonal antibodies (40). For example, low ERCC1 expression (common in Asian populations) is generally associated with better chemotherapy response to DNA-damaging agents like cisplatin (41) (42). Therefore, it is reasonable to assume that Asian populations may have higher drug exposure, potentially leading to more favorable outcomes when combining bsAbs with chemotherapy. Research has shown that the efficacy of certain bsAbs depends on immune function, which is influenced by age and physical status (43, 44). Our study demonstrated that in patients with an ECOG performance status of 1, bsAbs+chemotherapy demonstrated PFS benefits, suggesting better tolerance. However, caution is needed when making this inference, as OS was not affected. Conversely, in patients under the age of 65, bsAbs combined with chemotherapy showed OS benefits, indicating that this combination may be more suitable for frontline therapy. This age-related difference in outcomes may be partly explained by immune senescence, which can limit the effectiveness of these therapies in older patients. Immune senescence is characterized by a decline in immune function, including reduced T-cell diversity, impaired antigen presentation, and accumulation of senescent immune cells, all of which weaken the immune system’s ability to mount an effective anti-tumor response (45). In younger patients, a more robust immune system may better synergize with bsAbs and chemotherapy, enhancing tumor cell killing and prolonging survival. In contrast, older patients often exhibit a less responsive immune microenvironment, which may diminish the therapeutic benefits of bsAbs and chemotherapy combinations (46).

Subgroup analysis has also suggested that patients with solid tumors were more likely to receive survival benefits when treated with IgG-like bsAbs in combination with chemotherapy. Similar results were demonstrated in a nonrandomized controlled trial conducted by Birrer, M (47), who found that bintrafusp alfa, an IgG-like bsAbs, demonstrated clinical activity in patients with recurrent or metastatic cervical cancer. BsAbs are typically categorized into two types: IgG-like and non-IgG-like. IgG-like BsAbs are designed to mimic the structure of natural immunoglobulins (IgG), consisting of two heavy chains and two light chains. With a large molecular weight, IgG-like format containing Fc domains. Due to the presence of the Fc region, it can exhibit improving stability of the molecule and extending the half-life of the bsAbs, allowing for less frequent dosing (48, 49). Moreover, the Fc region is formed by the CH2 and CH3 domains of the heavy chains. It enables binding to Fc receptors on immune cells, facilitating ADCC and CDC (50). This dual mechanism enhances the immune system’s ability to target and eliminate tumor cells. In contrast, non-IgG-like bispecific antibodies lack the Fc region. They often consist of two single-chain variable fragments (scFvs) connected by a flexible peptide linker. Although absence of the Fc region leads to a shorter half-life, necessitating more frequent dosing, their smaller size allows for better tissue penetration, which can be advantageous in treating tumors with dense stroma or those located in hard-to-reach areas^35^. In summary, the choice between IgG-like and non-IgG-like bsAbs for solid tumor therapy depends on factors such as tumor type, location, and the desired immune response. Ongoing research aims to optimize these antibodies to balance tissue penetration with effective immune engagement (51). Taken together, there is still development space in bsAbs+chemotherapy application.

The overall safety of bsAbs+chemotherapy is acceptable as it did not increase the risk of most AEs involving the liver function, renal function, digestive system, and hematological system. Nevertheless, it’s important to note that adding bsAbs to chemotherapy does give rise to certain AEs that warrant attention. Leukopenia was significantly predisposed to occur in all grades. Since leukopenic individuals are more prone to severe, rapidly progressing infections that are often harder to treat, close monitoring of routine blood parameters following medication administration is essential. BsAbs+chemotherapy also increased the incidence of asthenia, weight decreased, and Infusion-related reaction, but these AEs can be effectively controlled by appropriated supportive care. The majority of AEs were driven by skin-related bsAbs toxic effects, such as dermatitis acneiform, paronychia, and rash, as well as reversible metabolic effects, including hypoproteinemia, hyperglycemia, and hypokalemia, often associated with chemotherapy. Nonetheless, these skin-related and metabolism related AEs are generally manageable with standard topical or systemic therapies. Intriguingly, clinical trials have shown that cancer patients who developed skin rash exhibited improved survival benefits compared with those without such skin reactions (52, 53). This underscores the possibility that immune-related skin rash might serve as a prognostic factor in patients with solid tumors. An alternative way to address the concerns of toxicity associated with bsAbs+chemotherapy may be to employ antibody-drug conjugates (ADCs), which induce less off-target toxicities by delivering cytotoxic payloads directly to tumor cells. Preclinical studies suggest that ADCs can induce immunogenic cell death (ICD), which enhances anti-tumor immune responses and may synergize with immunotherapy (54). However, research on the combination of ADCs with bsAbs remains limited (55), and further studies are needed to explore the potential synergies and safety profile of this approach. Overall, the AEs associated with bsAbs+chemotherapy are manageable but there is still a need for improvement and a necessity for close monitoring during therapy.

In our study, solid tumor was innovatively separated from the wide range of application areas of bsAbs+chemotherapy. Furthermore, efficacy and safety were analyzed from its components, targets and other multiple factors as well as multiple systems involving tumor and adverse reactions. As it should be, the limitation of this study was acknowledged. First, the data were aggregated at the study level instead of the individual level, which restricted our ability to examine more granular details. Additionally, the relatively small sample sizes within each subgroup may contribute to a reduction in the reliability of the results. This highlights the need for future research to involve multi-center, long-term RCTs to strengthen the evidence base.

## Conclusion

Generally, the combination of bsAb and chemotherapy could be a promising treatment option. Specifically, Asian patients, female patients, those under 65 years of age, and individuals treated with IgG-like bsAbs may benefit most from this combination. Meanwhile, potential toxicity on leukopenia, metabolism, and skin were also observed in patients, suggesting management of adverse events was of vital importance.

## Data Availability

The original contributions presented in the study are included in the article/**Supplementary Material**. Further inquiries can be directed to the corresponding authors.

## Supplementary material

Supplementary Figure 1 Risk of bias summary.

Supplementary Figure 2 Risk of bias graph.

Supplementary Figure 3 Funnel plots of PFS. The symmetrical distribution of the data points around the vertical line indicates no significant publication bias for PFS.

Supplementary Figure 4 Funnel plots of ORR. The symmetrical distribution of the data points around the vertical line suggests no significant publication bias for ORR.

Supplementary Figure 5 Funnel plots of OS. The slight asymmetry in the distribution of the data points suggests potential publication bias for OS.

Supplementary Table 1 Search Strategy.

Supplementary Table 2 Further Characteristics of the included trials.
