# Supplementary figures and images for: Bispecific antibodies combined with chemotherapy in solid tumor treatment, the path forward?
Source: Front Immunol. 2025 Apr 25;16:1568724. doi: 10.3389/fimmu.2025.1568724 (PMC12061958; doi:10.3389/fimmu.2025.1568724)

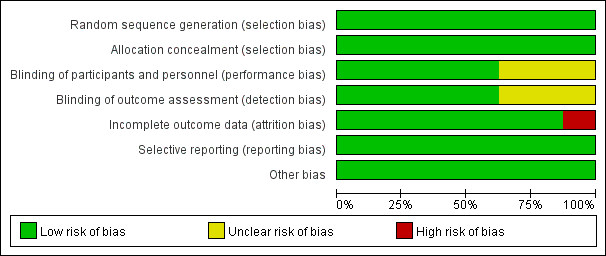

Supplement: Supplementary Figure 1 — Risk of bias summary. [file Image1.jpeg]

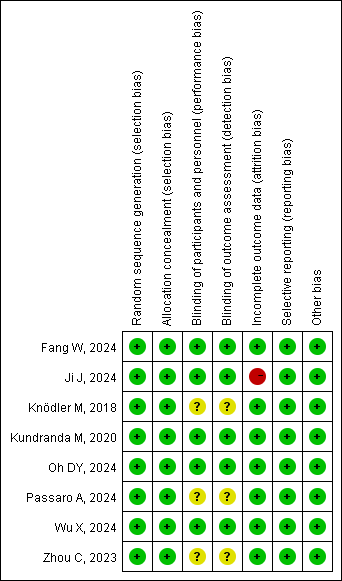

Supplement: Supplementary Figure 2 — Risk of bias graph. [file Image2.jpeg]

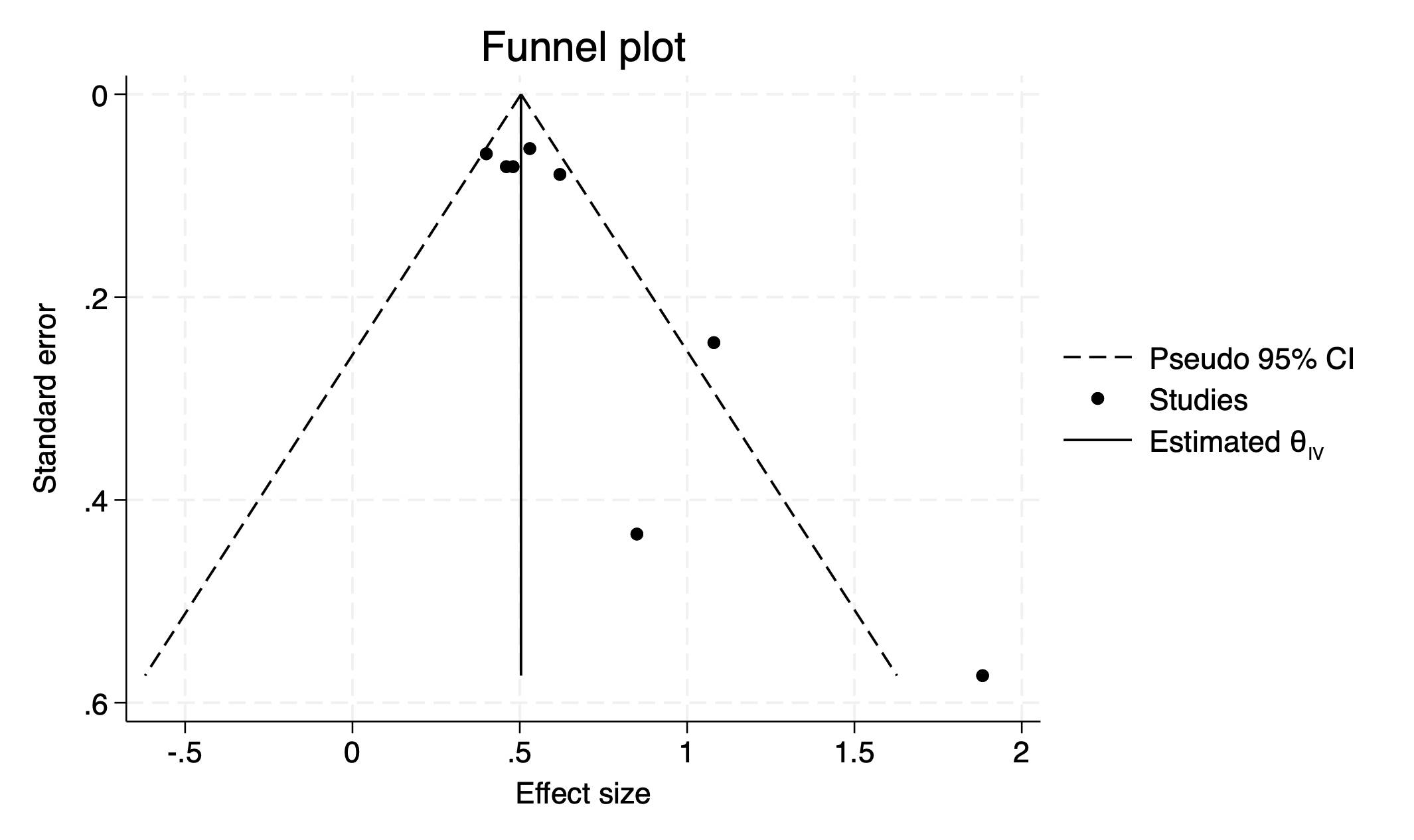

Supplement: Supplementary Figure 3 — Funnel plots of PFS. The symmetrical distribution of the data points around the vertical line indicates no significant publication bias for PFS. [file Image3.png]

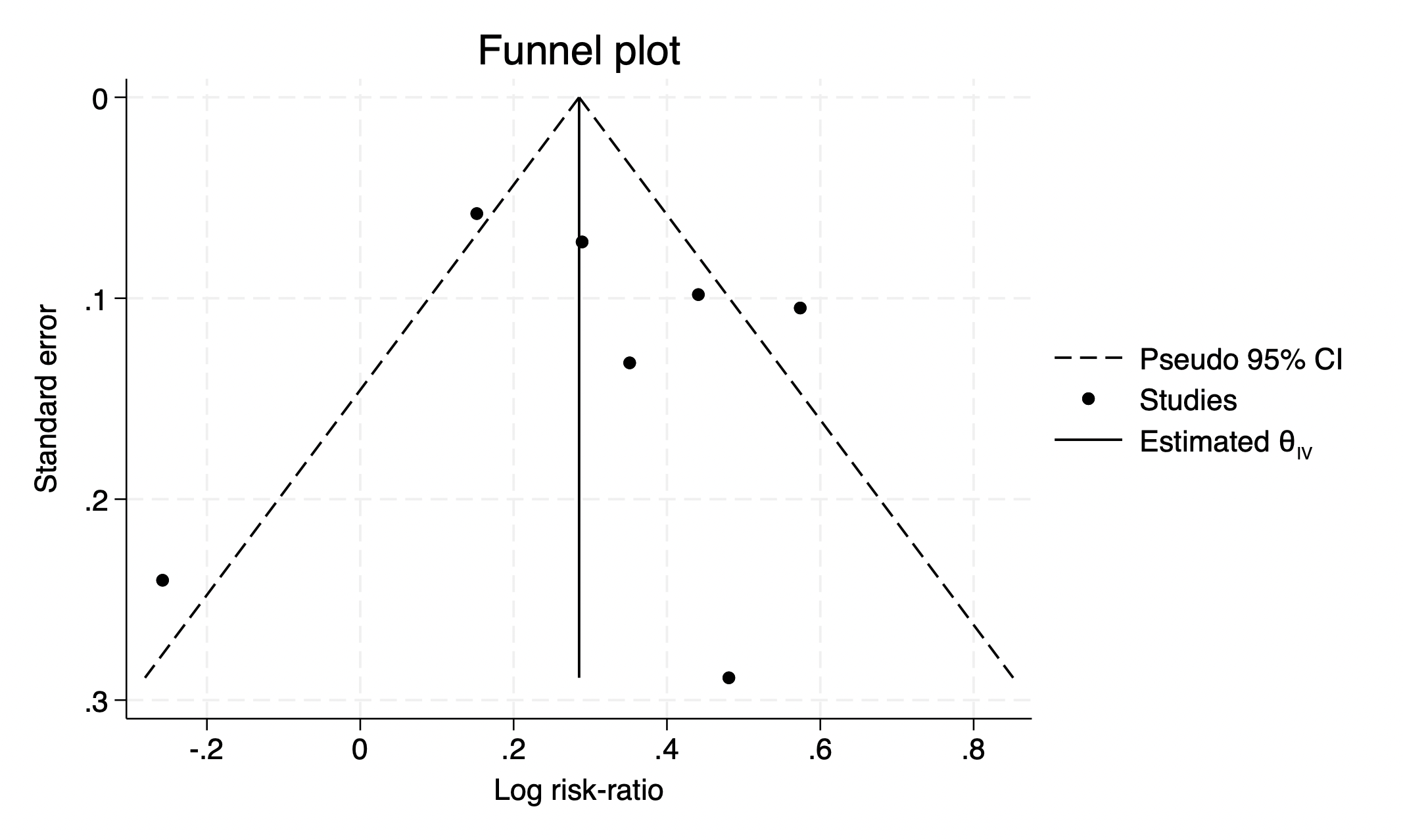

Supplement: Supplementary Figure 4 — Funnel plots of ORR. The symmetrical distribution of the data points around the vertical line suggests no significant publication bias for ORR. [file Image4.png]

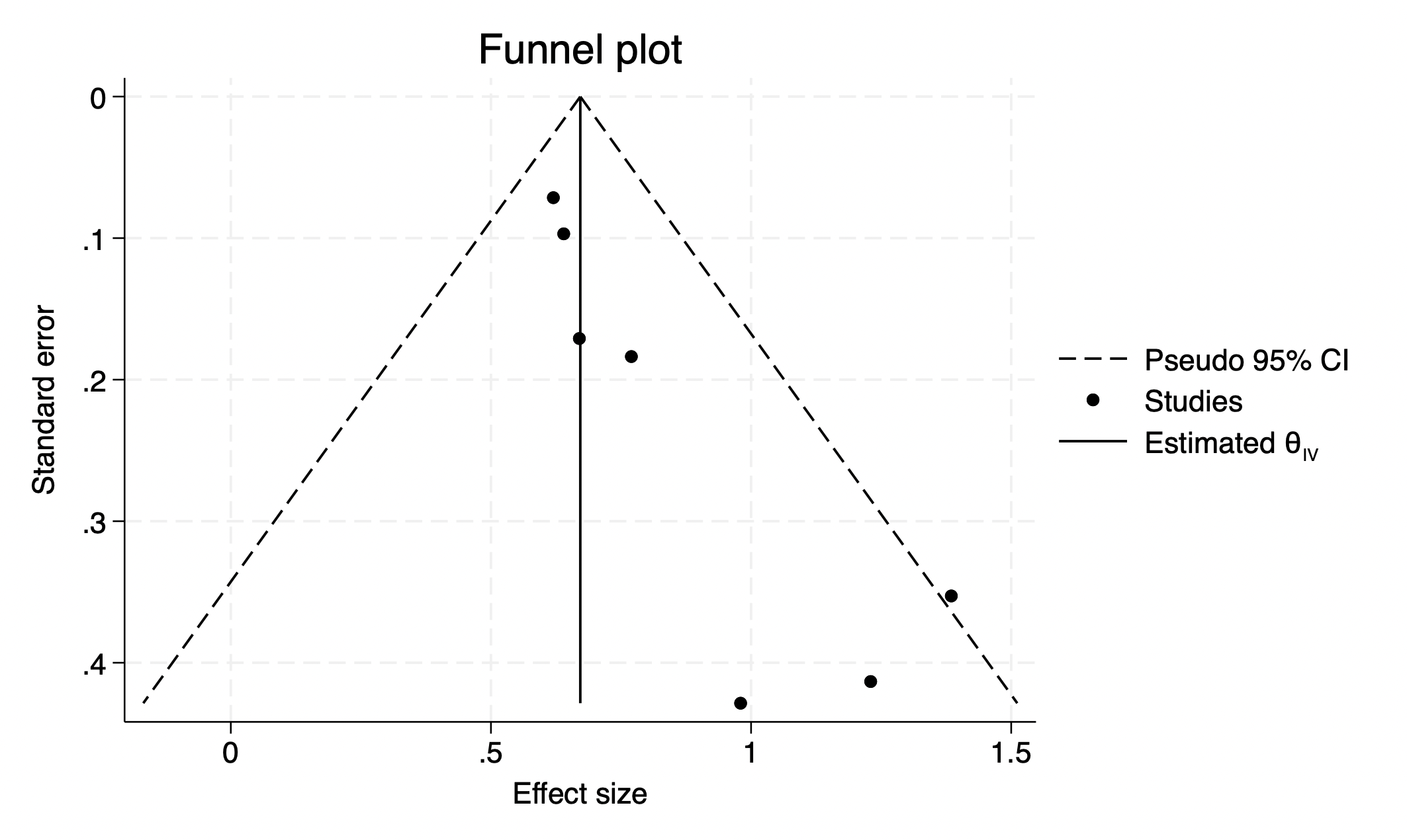

Supplement: Supplementary Figure 5 — Funnel plots of OS. The slight asymmetry in the distribution of the data points suggests potential publication bias for OS. [file Image5.png]
