# Supplementary material for: Bispecific antibodies combined with chemotherapy in solid tumor treatment, the path forward?
Source: Front Immunol. 2025 Apr 25;16:1568724. doi: 10.3389/fimmu.2025.1568724 (PMC12061958; doi:10.3389/fimmu.2025.1568724)
Supplement: Supplementary Table 1 — Search Strategy. [file Table1.docx]

**Supplementary Table 1. Search Strategy.**

**Data searched from PubMed**

| Search Query | Results |
| --- | --- |
| #1 "Antibodies, Bispecific"[Mesh] OR Bispecific Antibodies OR Bispecific Antibody OR Bispecific Monoclonal Antibodies OR Bifunctional Antibodies OR Catumaxomab OR Removab OR blinatumomab OR Blincyto OR amivantamab OR Rybrevant OR tebentafusp OR Kimmtrak OR mosunetuzumab OR BTCT-4465A OR Lunsumio OR cadonilimab OR AK104 OR teclistamab OR Tecvayli OR glofitamab OR Columvi OR talquetamab OR Talvey OR elranatamab OR Elrexfio OR epcoritamab OR Epkinly OR Odronextamab OR REGN1979 OR Linvoseltamab OR REGN5458 OR tarlatamab OR Imdelltra OR AMG 757 OR Erfonrilimab OR KN-046 OR Vudalimab OR XmAb20717 OR Volrustomig OR MEDI5752 OR Rilvegostomig OR AZD2936 OR Tebotelimab OR MGD013 OR Retlirafusp alfa OR SHR-1701 OR PM8002 OR ivonescimab OR AK112 OR Izalontamab OR SI-B001 OR CTX-009 OR Zenocutuzumab OR MCLA-128 OR Zanidatamab OR ZW25 OR Anbenitamab OR KN026 | 33,457 |
| #2 "Neoplasms"[Mesh] OR neoplas* OR tumor* OR cancer* OR malignan* OR tumour* OR carcinom* OR adenocarcin* | 6,015,911 |
| #3 ((((((((randomized controlled trial[Publication Type]) OR controlled clinical trial[Publication Type]) OR randomized[Title/Abstract]) OR placebo[Title/Abstract]) OR randomly[Title/Abstract]) OR "Clinical Trials as Topic"[Mesh:NoExp]) OR trial[Title])) NOT ((((animals[MeSH Terms]) NOT ((humans[MeSH Terms]) AND animals[MeSH Terms]))))))) | 1,536,195 |
| #4 #1 AND #2 AND #3 | 1,719 |

Searched before January 2, 2025. Results: 1,719.

**Data searched from** **Embase**

| Search Query | Results |
| --- | --- |
| #1 'bispecific antibody'/exp OR 'bispecific antibody' OR 'catumaxomab' OR 'blinatumomab' OR 'amivantamab' OR 'tebentafusp' OR 'mosunetuzumab' OR 'cadonilimab' OR 'teclistamab' OR 'glofitamab' OR 'talquetamab' OR 'elranatamab' OR 'epcoritamab' OR 'odronextamab' OR 'linvoseltamab' OR 'tarlatamab' OR 'erfonrilimab' OR 'vudalimab' OR 'volrustomig' OR 'rilvegostomig' OR 'tebotelimab' OR 'retlirafusp alfa' OR 'ivonescimab' OR 'izalontamab' OR CTX-009 OR 'zenocutuzumab' OR 'zanidatamab' OR 'anbenitamab' | 15,159 |
| #2 'neoplasm'/exp OR neoplas* OR tumor* OR cancer* OR malignan* OR tumour* OR carcinom* OR adenocarcin* | 8,667,368 |
| #3 'crossover procedure':de OR 'double-blind procedure':de OR 'randomized controlled trial':de OR 'single-blind procedured':de OR (random* OR factorial* OR crossover* OR cross NEXT/1 over* OR placebo* OR doubl* NEAR/1 blind* OR singl* NEAR/1 blind* OR assign* OR allocat* OR volunteer*):de,ab,ti | 3,458,945 |
| #4 #1 AND #2 AND #3 | 1,310 |

Searched before January 2, 2025. Results: 1,310.

**Data searched from Cochrane Library**

| Search Query | Results |
| --- | --- |
| #1 MeSH descriptor: [Antibodies, Bispecific] explode all trees | 119 |
| #2 (catumaxomab):ti,ab,kw OR (blinatumomab):ti,ab,kw OR (amivantamab):ti,ab,kw OR (tebentafusp):ti,ab,kw OR (mosunetuzumab):ti,ab,kw OR (cadonilimab):ti,ab,kw OR (teclistamab):ti,ab,kw OR (glofitamab):ti,ab,kw OR (talquetamab):ti,ab,kw OR (elranatamab):ti,ab,kw OR (epcoritamab):ti,ab,kw OR (odronextamab):ti,ab,kw OR (linvoseltamab):ti,ab,kw OR (tarlatamab):ti,ab,kw OR (erfonrilimab):ti,ab,kw OR (vudalimab):ti,ab,kw OR (volrustomig):ti,ab,kw OR (rilvegostomig):ti,ab,kw OR (tebotelimab):ti,ab,kw OR (retlirafusp alfa):ti,ab,kw OR (ivonescimab):ti,ab,kw OR (izalontamab):ti,ab,kw OR (CTX-009):ti,ab,kw OR (zenocutuzumab):ti,ab,kw OR (zanidatamab):ti,ab,kw OR (anbenitamab):ti,ab,kw | 584 |
| #3 MeSH descriptor: [Neoplasms] explode all trees | 128,820 |
| #4 (cancer):ti,ab,kw OR (tumor):ti,ab,kw OR (adenocarcinoma):ti,ab,kw | 250,972 |
| #5 #1 OR #2 | 645 |
| #6 #3 OR #4 | 281,765 |
| #7 #5 AND #6 | 531 |

Searched before January 2, 2025. Results: 531.
