# Supplementary material for: Bispecific antibodies combined with chemotherapy in solid tumor treatment, the path forward?
Source: Front Immunol. 2025 Apr 25;16:1568724. doi: 10.3389/fimmu.2025.1568724 (PMC12061958; doi:10.3389/fimmu.2025.1568724)
Supplement: Supplementary Table 2 — Further Characteristics of the included trials. [file Table2.docx]

**Supplementary Table 2. Characteristics of included articles.**

| Author, year | bsAb formats | Masking | Phase | No. of male | Target |
| --- | --- | --- | --- | --- | --- |
| Knödler M, 2018 | Non-IgG-like | open-label | 2 | 17 | EpCAM×CD3s |
| Kundranda M, 2020 | IgG-like | double-blind | 2 | 46 | IGF-1R×ErbB3 |
| Zhou C, 2023 | IgG-like | open-label | 3 | 130 | c-Met×EGFR |
| Ji J, 2024 | IgG-like | double-blind | 3 | 474 | PD1×CTLA4 |
| Fang W, 2024 | IgG-like | double-blind | 3 | 156 | PD1×VEGF-A |
| Oh DY, 2024 | Non-IgG-like | double-blind | 2/3 | 151 | PD-L1×TGF-β |
| Passaro A, 2024 | IgG-like | open-label | 3 | 238 | c-Met×EGFR |
| Wu X, 2024 | IgG-like | double-blind | 3 | 0 | PD1×CTLA4 |

EpCAM, epithelial cell adhesion molecule; IGF-1R, insulin-like growth factor 1 receptor; ErbB3, epidermal growth factor receptor 3; c-Met, mesenchymal-epithelial transition factor; EGFR, epidermal growth factor receptor; PD1, programmed cell death protein 1; CTLA4, cytotoxic T-lymphocyte-associated protein 4; VEGF-A, vascular endothelial growth factor A; PD-L1, programmed cell death-ligand 1; TGF-β, transforming growth factor beta; bsAb, bispecific antibody.
