# Supplementary material for: Bispecific antibodies combined with chemotherapy in solid tumor treatment, the path forward?
Source: Front Immunol. 2025 Apr 25;16:1568724. doi: 10.3389/fimmu.2025.1568724 (PMC12061958; doi:10.3389/fimmu.2025.1568724)
Supplement: Supplementary file 8 [file Table3.docx]

**Supplementary Table 3.** Subgroup analysis of Metabolism adverse effect stratified by chemotherapy.

| Adverse events | | RR (95% CI) | | | | | |
| --- | --- | --- | --- | --- | --- | --- | --- |
|  |  | No. of studies | All Grade | P value | No. of studies | Grade≥3 | P value |
| HYPE | CP | 2 | 1.91 (1.07, 3.40) | 0.028 | NA | NA | NA |
| HypoK | CP | 2 | 1.83 (0.70, 4.80) | 0.219 | 2 | 1.52 (0.61, 3.76) | 0.366 |

RR, relative risk, NA, not available; HYPE, hypoproteinemia; HypoK, hypokalemia; CP, carboplatin plus pemetrexed regimen
